# Secretion of biologically active pancreatitis-associated protein I (PAP) by genetically modified dairy *Lactococcus lactis* NZ9000 in the prevention of intestinal mucositis

**DOI:** 10.1186/s12934-017-0624-x

**Published:** 2017-02-13

**Authors:** Rodrigo D. Carvalho, Natalia Breyner, Zelia Menezes-Garcia, Nubia M. Rodrigues, Luisa Lemos, Tatiane U. Maioli, Danielle da Gloria Souza, Denise Carmona, Ana M. C. de Faria, Philippe Langella, Jean-Marc Chatel, Luis G. Bermúdez-Humarán, Henrique C. P. Figueiredo, Vasco Azevedo, Marcela S. de Azevedo

**Affiliations:** 1 Federal University of Minas Gerais (UFMG-ICB), Av. Antônio Carlos, 6627, CP 486, Belo Horizonte, MG 31270-901 Brazil; 2 Micalis Institute, INRA, AgroParisTech, Université Paris-Saclay, 78350 Jouy-En-Josas, France

**Keywords:** Pancreatitis-associated protein, Mucositis, *Lactococcus lactis*, 5-Fluoracil

## Abstract

**Background:**

Mucositis is one of the most relevant gastrointestinal inflammatory conditions in humans, generated by the use of chemotherapy drugs, such as 5-fluoracil (5-FU). 5-FU-induced mucositis affects 80% of patients undergoing oncological treatment causing mucosal gut dysfunctions and great discomfort. As current therapy drugs presents limitations in alleviating mucositis symptoms, alternative strategies are being pursued. Recent studies have shown that the antimicrobial pancreatitis-associated protein (PAP) has a protective role in intestinal inflammatory processes. Indeed, it was demonstrated that a recombinant strain of *Lactococcus lactis* expressing human PAP (LL-PAP) could prevent and improve murine DNBS-induced colitis, an inflammatory bowel disease (IBD) that causes severe inflammation of the colon. Hence, in this study we sought to evaluate the protective effects of LL-PAP on 5-FU-induced experimental mucositis in BALB/c mice as a novel approach to treat the disease.

**Results:**

Our results show that non-recombinant *L. lactis* NZ9000 have antagonistic activity, in vitro, against the enteroinvasive gastrointestinal pathogen *L. monocytogenes* and confirmed PAP inhibitory effect against Opportunistic *E. faecalis.* Moreover, *L. lactis* was able to prevent histological damage, reduce neutrophil and eosinophil infiltration and secretory Immunoglobulin-A in mice injected with 5-FU. Recombinant lactococci carrying antimicrobial PAP did not improve those markers of inflammation, although its expression was associated with villous architecture preservation and increased secretory granules density inside Paneth cells in response to 5-FU inflammation.

**Conclusions:**

We have demonstrated for the first time that *L. lactis* NZ9000 by itself, is able to prevent 5-FU-induced intestinal inflammation in BALB/c mice. Moreover, PAP delivered by recombinant *L. lactis* strain showed additional protective effects in mice epithelium, revealing to be a promising strategy to treat intestinal mucositis.

## Background

Mucositis is a gastrointestinal inflammatory disorder caused by radiotherapy or chemotherapeutic agents in oncology patients [1–3]. This enfeebling condition has been reported in 80% of patients undergoing clinical treatment with 5-fluorouracil (5-FU), a drug commonly prescribed for treating several types of cancer, including gastrointestinal, breast, pancreas, head and neck [2, 4]. As 5-FU present nonspecific cytotoxicity to cells with high replication rate, it inhibits the proliferation of both cancer and normal cells, such as the enterocytes lining the digestive tract [5, 6]. Hence, this process often leads to mucosal alterations, characterized by leukocyte infiltration, ulcers, villus shortening and decreased villus/crypt ratio, favoring systemic translocation of harmful bacteria colonizing the gut, increasing thus the possibility of infections and fatal consequences [7–11]. Moreover, 5-FU-induced mucositis has been associated with mucosal gut dysfunctions, affecting patients’ food consumption, provoking vomiting, abdominal pain, and diarrhea which may cause dehydration [1, 3].

Up to date, mucositis treatment relies mostly on mucosal coatings, cryotherapy, antibiotics and analgesics administration. However, no current therapy has been efficient to alleviate the disease [12, 13]. Thus, alternative strategies are currently being investigated. Contemporary studies have described promising achievements with the administration of lactic acid bacterium (LAB), instead of drugs [14–18]. These bacteria are considered to be probiotics, a term defined as “live microorganisms administered in adequate amounts that confer a beneficial health effect on the host” [19].

Species like *Streptococcus thermophilus* and several *Lactobacilli* have been shown to reduce intestinal inflammation caused by chemotherapy drugs injection, such as 5-FU, irinotecan or methotrexate, in rats [14–16]. Moreover, administration of yogurts containing *L. johnsonii* or *L. bulgaricus* and *S. thermophilus*, seems to be useful to restore intestinal barrier function in these animals [17].

The use of recombinant LAB strains, such as the model *Lactococus lactis* for delivering biologically active molecules with anti-inflammatory properties has also been explored as an alternative therapy for the treatment of mucositis and other gastrointestinal inflammatory disorders, as inflammatory bowel diseases (IBD) [20–23]. In 2006, a biological confinement strategy to contain the dissemination of genetically engineered *L. lactis* strain expressing IL-10 was carried out in phase I clinical trial with IBD patients, suggesting the feasibility of mucosal therapy using recombinant lactococci [24]. This trial provided novel possibilities for testing genetically modified *L. lactis* to treat similar intestinal disorders, such as mucositis. In fact, recent studies have designed recombinant strains of *L. lactis* to produce anti-inflammatory proteins, involved in the maintenance of epithelial barrier integrity, such as Trefoil factor 1 (TFF-1), which has been promising to treat mucositis in clinical trial as well [25, 26]. Therefore, other key elements in the host-microbiota relationship are being glimpsed as potential candidates to be cloned in *L. lactis.*

Delivery of antimicrobial peptides (AMP) that protects the host by killing harmful bacteria has been shown to prevent inflammation in colitis mice models [27, 28]. This positive effect was associated with host epithelial cell surface protection against pro-inflammatory bacteria in the intestinal mucosa [27, 28]. As AMP have been recently considered to be a very effective approach to fight inflammation, various types are being explored in basic research such as Reg3A, also known as pancreatitis associated protein (PAP). It has been extensively studied due to its protective effect in the intestinal inflammatory process [29–33]. This protein belongs to the RegIII gene sub-family, which encodes proteins involved in the regulation of epithelial cell proliferation and antimicrobial activity in several organs, including intestines [29–32]. In the small intestine, PAP is mainly produced by Paneth cells that are located in mucosal crypts and exerts bactericidal activity against Gram-positive bacteria species that might pose risk of infection to the host [32, 33]. Recently, our research group, constructed and confirmed the expression of human PAP by recombinant *L. Lactis* NZ9000 using the inducible Nisin Control Expression System (NICE) [34]. This work evaluated the therapeutic effect of this strain in mice model of dinitrobenzenosulfonic acid (DNBS)-induced colitis. We found out that PAP delivered by lactococci revealed to be anti-inflammatory [34]. As this strategy have shown to be useful in the treatment of IBD, we sought to investigate LL-PAP protective role in another important inflammatory gastrointestinal disorder for which conventional therapy is not enough, as mucositis, using the 5-FU intestinal mucositis experimental mouse model.

## Methods

### Bacterial strains and growth conditions

*Lactococcus lactis* NZ9000 strain harboring pSEC:PAP vector (LL-PAP) and *L. lactis* NZ9000 strain [35] carrying pSEC vector without the open reading frame of PAP (LL) [36], were grown in M17 medium (Difco) supplemented with 0.5% glucose (GM17) at 30 °C without shaking. Recombinant strains were selected by the addition of chloramphenicol (Cm, 10 µg/mL). For nisin-induced PAP expression, LL-PAP was cultivated until the optical density at 600 nm reached 0.6. Afterwards, 10 ng/mL of nisin (Sigma) were added to the medium and cultures were maintained at 30 °C for 2 h before experimentation. For in vitro antagonistic assays, *L. lactis* strains were grown in brain–heart infusion (BHI) medium containing Cm (10 µg/mL) at 30 °C, as well as *Listeria monocytogenes* ATCC 15313 and *Enterococcus faecalis* ATCC 19433, which was grown in BHI containing or not Cm (10 µg/mL) at 37 °C without shaking.

### Antagonistic activity assay against pathogenic *Listeria monocytogenes and Enterococcus faecalis*

Antimicrobial activity of PAP secreted by LL-PAP strain was assessed against the food-borne pathogen *L. monocytogenes* or the commensal opportunistic *E. faecalis* using a previously described method [37]. Briefly, LL and LL-PAP were grown separately in BHI medium containing Cm (10 µg/mL) and, after OD600 reached 0.6, 10 ng/mL of nisin were added. Cultures were then centrifuged in an OD600 of 1.0, at 4000 *g* for 10 min and supernatants were sterilized using 0.20 µm Millipore filters (Sarstedt, Nümbrecht, Germany). *L. monocytogenes* or *E. faecalis* were then inoculated separately into filter-sterilized lactococcal supernatant or in BHI medium containing Cm (10 µg/mL) and nisin (10 ng/mL) at an initial OD600 of 0.1. Cultures were incubated at 37 °C and after 2 and 4 h respectively, serial dilutions were seeded in BHI agar plates that were maintained at 37 °C for 24 h. Thereafter, number of viable *L. monocytogenes or F. faecalis* was estimated through counting of bacterial colony forming units (CFU).

### Animals, bacterial administration and experimental groups

Conventional female BALB/c mice between 6 and 8 weeks of age were obtained at Federal University of Minas Gerais (UFMG–Belo Horizonte, Brazil) and the study was approved by the Brazilian Ethics Committee on Animal Use (CEUA). Mice were kept in a temperature-controlled room with ad libitum access to water and standard chow diet 24 h prior to experiments. Animals were fed daily orally by drinking 5 mL of water; or 5 mL of M17 medium supplemented with Cm (10 µg/mL) and nisin (10 ng/mL) (M17/Cm/Nisin); or with 5 mL of M17/Cm/Nisin containing 2.5 × 10^9^ CFU/mL of either LL or LL-PAP strains for 13 days. In order to induce mucositis, mice received a single intraperitoneal injection of 5-fluoracil (300 mg/kg) on day 10 and were euthanized on day 14. An injection of saline (NaCl 0.9%) was used as a control. For experimentation, BALB/c mice were divided into eight groups, each containing 6–9 animals. Animals from group 1–4 were injected with 0.9% saline on day 10 (noninflamed groups); group 1 received water, group 2 were fed with M17/Cm/Nisin medium; group 3 were administered with LL culture, group 4 received *L. lactis* expressing PAP. Mouse from group 5–8 were injected with 5-FU on day 10 (inflamed groups): group 5 received water; group 6 received M17/Cm/Nisin medium; group 7 were administered with LL culture and finally group 8 received LL-PAP strain.

### Intestinal histology and morphology

After euthanasia, the distal portion of the small bowel (ileum) from the animals was collected, and, after washing, rolls were prepared for histomorphological analysis. Rolls were fixed with 10% buffered formaldehyde. Material was then embedded in paraffin, and a 4 µm section of each sample was placed on a glass slide and stained with hematoxylin and eosin (HE). Slides of each experimental group were photographed using a digital camera (Moticam 2500, China) coupled to an optical microscope (Olympus Optical Co., Japan). The histological score was determined using a previously described method [10], which measures the intensity of both mononuclear and polymorphonuclear cells infiltrate in the *lamina propria*, changes in mucosal architecture and presence of ulceration. For each parameter it was used the ranking values: absent (0), mild (1), moderate (2) and severe (3). For morphological analysis, ten images from the ileum of each animal were randomly captured and analyzed through ImageJ software. Granular density inside Paneth cells was determined by measuring the intracellular area occupied by secretory granules. Villus height and crypt depth was measured vertically from the tip of a villus to the base of the adjacent crypt. Villus height⁄crypt height ratio from the intestinal epithelium was also obtained.

### Determination of intestinal myeloperoxidase and eosinophil peroxidase activity

The extent of neutrophil accumulation in the small bowel was assessed by determination of myeloperoxidase (MPO) activity, as described previously [38]. Briefly, a 3 cm portion of mice intestines were removed and 100 mg of intestine were weighted and homogenized with 1 mL of PBS and centrifuged at 12,000*g* for 10 min. The supernatant was discarded, and the erythrocytes were lysed. The samples were then centrifuged, the supernatant was discarded, and the pellet was suspended in 1 mL of 0.05 M Na_3_PO_4_, frozen three times in liquid nitrogen, and centrifuged at 4 °C at 12,000*g* for 10 min. The supernatant was used in the enzymatic assay by the addition of an equal amount substrate (2.9 mmol of tetramethylbenzidine in DMSO). The reaction was stopped with 50 µL of 1 M H_2_SO_4_, and the absorbance was read at 450 nm. Results were expressed as the relative unit that denotes activity of MPO related with casein-elicited murine peritoneal neutrophils processed in the same way.

The extent of tissue eosinophil infiltration was assessed by measurement of eosinophil peroxidase (EPO) activity, as previously described [39]. Briefly, 100 mg of intestine were weighted, homogenized with 1.9 mL of PBS and centrifuged at 12,000*g* for 10 min. Supernatant was discarded, and pellet was resuspended in 1.9 mL of 0.5% hexadecyltrimethyl ammonium bromide diluted in PBS. After being frozen three times in liquid nitrogen, samples were centrifuged at 4 °C, 12,000*g* during 10 min. To test EPO activity, the obtained supernatant was mixture with a substrate (1:1) containing 1.5 mmol/L of o-phenylenediamine, 6.6 mmol/L of H_2_O_2_ and 0.075 mmol/L of Tris–HCl (pH 8). Reaction was stopped with 50 µL of 1 M H_2_SO_4_, and absorbance was measured at 492 nm.

### Secretory IgA

Levels of secretory IgA were determined by enzyme linked immunosorbent assay (ELISA) in small bowel intestinal fluids. Microtitre plates (Nunc-Immuno Plates, MaxiSorp) were coated with goat anti-mouse antibody (Southern Biotechnology, Birmingham, AL, USA) in carbonate-bicarbonate buffer (0.1 M Na2CO3/NaHCO3—pH 9.6) for 18 h at 4 °C. Wells were washed with washing solution (saline 0.9% plus 0.05% tween 20) and blocked with 200 µL of 0.05% casein in PBS for 1 h at room temperature. Intestinal fluids previously centrifuged at 432*g* for 20 min were added to the plate and diluted in PBS-0.25% casein (two times until dilution 1:80). After incubation of 1 h at room temperature, plate was washed and biotin conjugated anti-mouse IgA antibody (Southern Biotechnology) in PBS-0.25% casein (1:10.000) was added to the wells. After incubation of 1 h at 37 °C, peroxidase-streptavidin goat anti-mouse IgA (Southern Biotechnology, Birmingham, AL, USA) was added; plate was incubated for 1 h more and, then, coated with 100 µL/well of orthophenylenediamine (OPD) (1 mg/mL) (Sigma, St. Louis, MO, USA) and 0.04% H_2_O_2_ substrates. Color was developed at room temperature and reaction was stopped by the addition of 20 µL/well of 2 N H_2_SO_4_. Absorbance was measured at 492 nm using a Bio-Rad Model 450 Microplate Reader. Results were expressed as concentration (µg/mL), according to the standard curve.

### Statistical analysis

Differences between groups were statistically evaluated by one way analysis of variance (ANOVA). Bonferroni test was applied to calculate statistical significance across groups. Non-parametric Mann–Whitney test was used for data sets based on scores or percentages. All data was processed using GraphPad Prism 5.0 software. P values under 0.05 were considered significant.

## Results

### *L. lactis* supernatant inhibits in vitro growth of *L. monocytogenes*

**Fig. 1:**
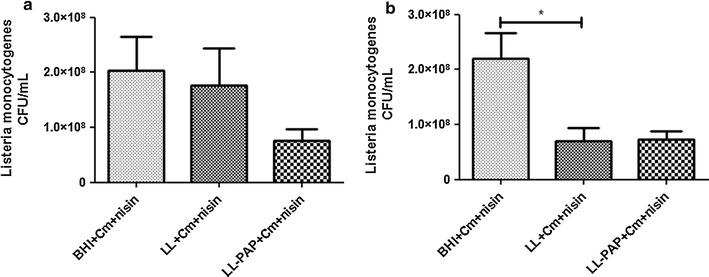
Inhibitory activity of bacterial supernantants from *L. lactis* and *L. lactis*-PAP against pathogenic *L. monocytogenes*, after 2 (**a**) and 4 h (**b**) incubation. *p < 0.05

The antagonistic activity of LL or LL-PAP culture supernatants was assessed against the food-borne pathogen *L. monocytogenes.* After 2 h, it was not observed any statistical differences in CFU counts of *L. monocytogenes* inoculated in BHI + Cm + Nisin medium or in the supernatant of LL and LL-PAP, both containing Cm and Nisin (Fig. 1a). However, after 4 h, it was observed a twofold decrease in CFU counts of *L. monocytogenes* grown either in LL + Cm + Nisin or in LL-PAP + Cm + Nisin culture supernatants when compared to counts obtained for the pathogen inoculated in BHI + Cm + Nisin medium. Presence of PAP in the supernatant did not reduce *L. monocytogenes* counts (Fig. 1b).

### PAP-producing *L. lactis* strain supernatant inhibits in vitro of *E. faecalis*

**Fig. 2:**
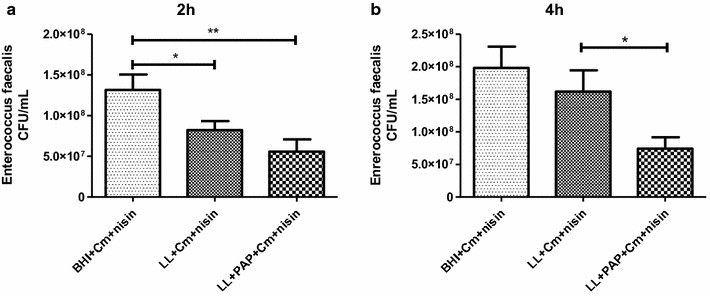
Inhibitory activity of bacterial supernantants from *L. lactis* and *L. lactis*-PAP against pathogenic *E. faecalis*, after 2 (**a**) and 4 h (**b**) incubation. **p < 0.003; *p < 0.05

The inhibitory activity of LL or LL-PAP culture supernatants was evaluated against the opportunistic commensal *E. faecalis.* After 2 h, it was observed a statistical differences in CFU counts of *E. faecalis* inoculated in BHI + Cm + Nisin medium compared to supernatant of both LL and LL-PAP cultures, 1.6- and 2-fold respectively (Fig. 2a). Moreover, after 4 h, it was observed a 2.2-fold decrease in CFU counts of *E. faecalis* grown in LL-PAP + Cm + Nisin culture supernatants when compared to counts obtained for the enterococci inoculated in LL + Cm + Nisin culture. LL supernatant did not reduce *E. faecalis* counts after 4 h (Fig. 2b).

### *L. lactis* administration prevents 5-FU-induced mucosal inflammation in the ileum

**Fig. 3:**
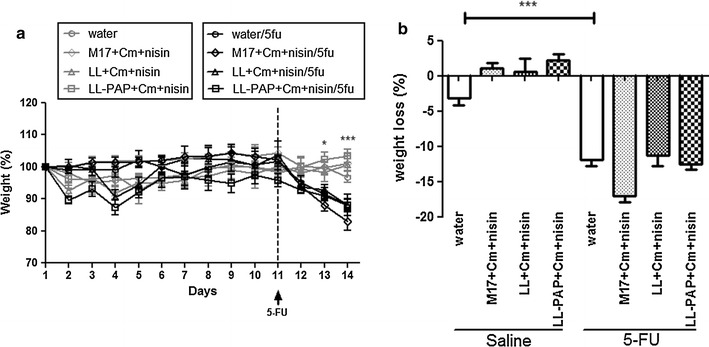
Time-course of body weight for mice injected with saline or 5-fluoracil receiving water, M17 + Cm + Nisin medium or *L. lactis* and *L. lactis*-PAP strains (**a**). Weight loss observed after 5-FU injection and differences across groups (**b**).*p < 0.05; ***p < 0.0001

**Fig. 4:**
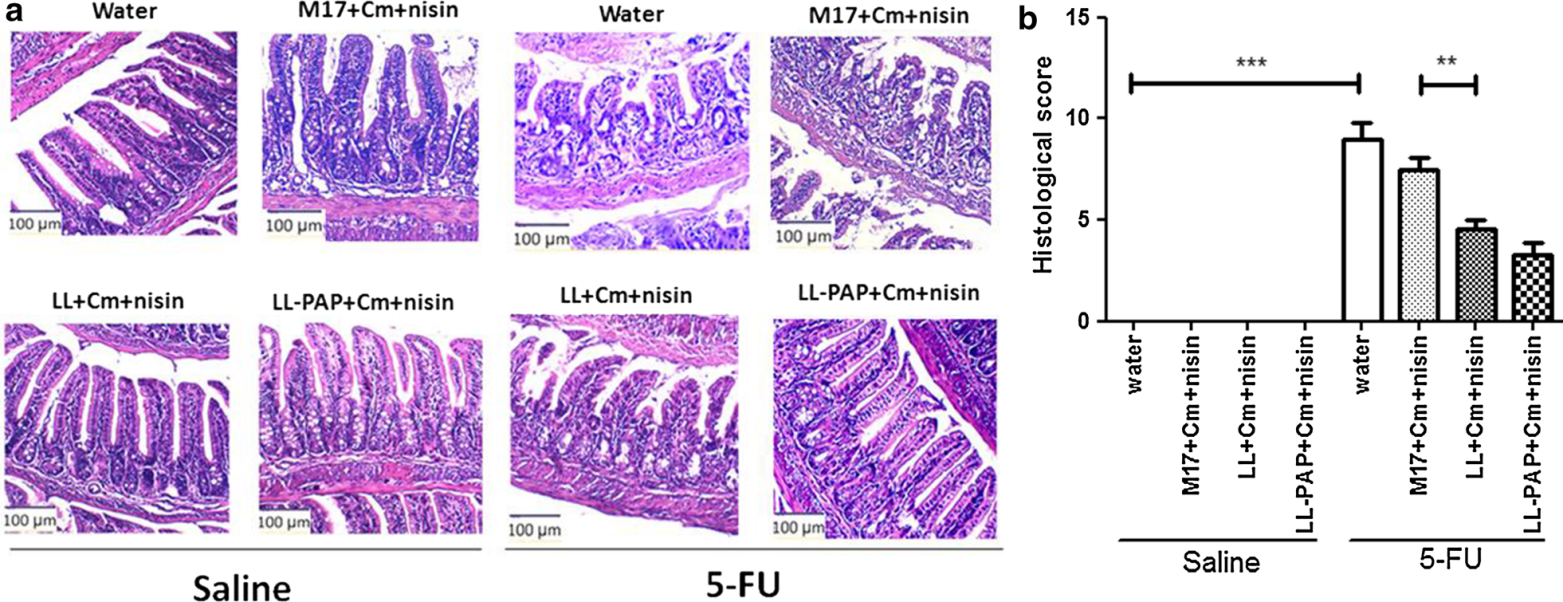
Representative images from mucosal histopathology (**a**) and histopathological scores obtained for experimental groups (**b**). ***p < 0.0001; **p < 0.003

Mice injected with 5-FU showed significant decreases in body weight (10 and 17% loss) from groups fed with water or M17 + Cm + Nisin medium as expected (Fig. 3). Treatment with *L. lactis* strains did not improve mouse weight as groups receiving LL or LL-PAP cultures lost approximately 10% of their initial body weight after 5-FU injection (Fig. 3b). Histological analysis revealed mucosal pattern within normal limits in all groups injected with 0.9% saline (Fig. 4a). 5-FU injection in mice receiving water or M17 + Cm + Nisin medium caused lesions in the small intestine characterized by an inflammatory cellular infiltrate in *lamina propria*, as well as in the submucosa and muscular layers, with increased number of histopathological parameters (Fig. 4b). Mice immunized with LL prevented 5-FU-induced mucosal inflammation in the ileum, showing reduced infiltration by polymorphonuclear neutrophils, ulceration and reduced alterations of intestinal mucosal architecture. Treatment with LL-PAP strain did not improve the histological score (Fig. 4b).

### Delivery of human PAP by *L. lactis* improves villous architecture preservation and Paneth cells activity in ileum

**Fig. 5:**
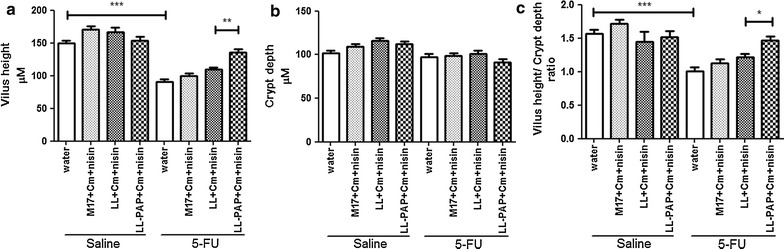
Morphometric analysis of villus height (**a**), crypts depth (**b**) and villus height/crypt depth ratio (**c**). ***p < 0,0001; **p < 0.003; *p < 0.005

**Fig. 6:**
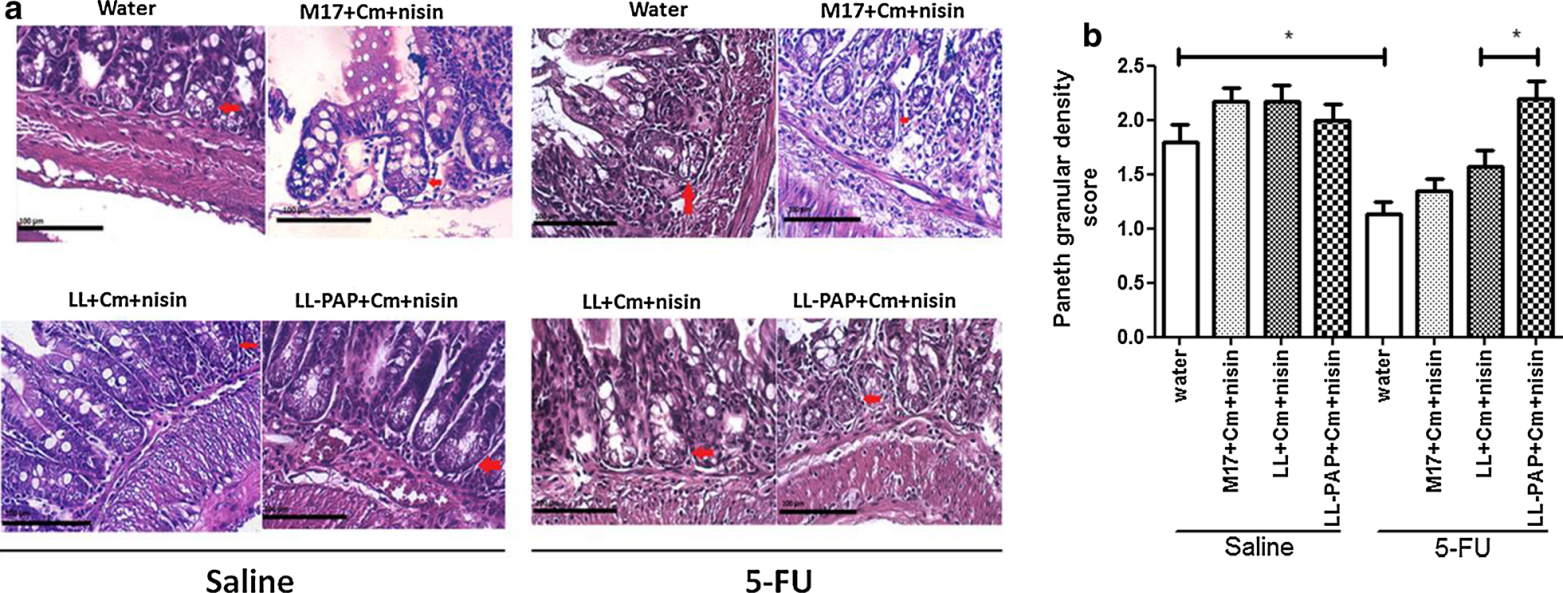
Representative images from Paneth cells morphology (**a**) and microscopic morphometric analysis of Paneth cell secretory granules (**b**). *p > 0.05

Morphometric analysis was carried out to evaluate epithelial integrity. A decrease was observed in villus height (Fig. 5a), villus/crypt ratio (Fig. 5c) and granular density inside Paneth cells (Fig. 6) after 5-FU injection in mice receiving water or M17 + Cm + Nisin medium. No statistical differences in crypt depth were observed across groups (Fig. 5b). Inflamed mice treated with LL-PAP showed increased villus height (Fig. 5a), vilus/crypt ratio (Fig. 5c) and granular density within Paneth cells (Fig. 6a, b) when compared to mice treated with LL.

### *L. lactis* treatment reduces neutrophilic and eosinophilic infiltration in the ileum

**Fig. 7:**
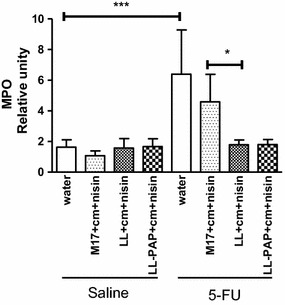
Differences across experimental groups in myeloperoxidase activity from cell lysates of the ileum. ***p < 0.0001; *p > 0.05

**Fig. 8:**
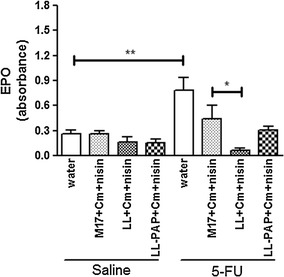
Differences across experimental groups in eosinophil peroxidase activity from cell lysates of the ileum. **p < 0.003; *p > 0.05

To evaluate whether treatment with LL or LL-PAP strain would have an effect in reducing polymorphonuclear cells infiltration in the small bowel of mice, MPO and EPO activity were measured in cell lysates of the ileum. As expected, 5-FU administration increased both intestinal MPO and EPO activity in mice administered with water or M17 + Cm + Nisin medium (Figs. 7, 8). Treatment with LL culture demonstrated to reduce infiltration those enzymes activity when compared to mice that were given M17 + Cm + Nisin medium. Expression of PAP by *L. lactis* did not reduce neither EPO nor MPO activity (Figs. 7, 8).

### *L. lactis* reduces IgA secretion in the small bowel

**Fig. 9:**
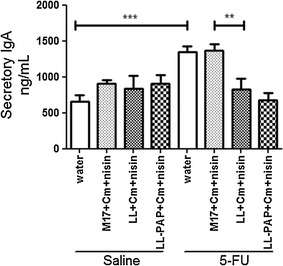
Evaluation of IgA secretion in the small bowel from inflamed and noninflamed mice receveing water, M17 medium or *L. lactis* and *L. lactis*-PAP strains. ***p < 0.0001; **p < 0.003

Secretory IgA (sIgA) response was also measured in the small bowel of mice as it plays an important role in mucosal protection. sIgA secretion increased in mice injected with 5-FU receiving water or M17 + Cm + Nisin medium, as expected (Fig. 9). Group of animals immunized with LL or LL-PAP strain demonstrated reduced levels of IgA when compared to M17-treated group (Fig. 9). However, LL-PAP administration did not caused a significant reduction of sIgA when compared to LL-treated mice (Fig. 9).

## Discussion

PAP anti-microbial role against Gram-positive pathogenic bacteria has been demonstrated in previous studies [29–33]. Hence, we decided to investigate whether recombinant PAP expressed by *Lactococcus lactis* would have an inhibitory in vitro effect against Gram-positive foodborne pathogen *Listeria monocytogenes* and opportunistic commensal *E. faecalis*. Interestingly, we found that the bacterial supernatant from either *L. lactis* NZ9000 or *L. lactis* expressing PAP cultures could statistically inhibit both *L. monocytogenes and E. faecalis* growth. Moreover, expression of PAP seems to enhance this inhibitory effect against *E. faecalis*. In our work we did not add trypsin, which is necessary for PAP N-terminal cleavage and activation [31–33], to the medium as a recent work demonstrated that intact unprocessed PAP protein was capable of binding and killing Gram-positive bacteria [29, 30]. Indeed, although we used non-concentrated supernatant from LL-PAP, it inhibited *E. faecalis* growth revealing PAP anti-microbial activity against this Gram-positive species. Moreover, *L. lactis* antagonism against both pathogens could be caused by expression of some molecules as lactic acid, bacteriocins or metabolites naturally produced by this LAB species. Actually, Charlier and colleagues [40] had tested the inhibitory effect of seventy-five strains of *L. lactis*, including *L. lactis* NZ9000 strain, against Gram-positive pathogenic *Staphylococcus aureus.* They demonstrated that medium acidification was not involved in the inhibition of *S. aureus* in early growth phases, suggesting that further experimentation is required to characterize the molecular bases of *L. lactis* NZ9000 antagonistic activity against this pathogen [40].

Besides its antimicrobial activity, PAP has been reported to be involved in maintaining intestinal homeostasis [32]. Indeed, a recent report from our research group showed that *L. lactis* expressing PAP was capable to efficiently prevent colitis in mice [34]. Therefore, we supposed that this strain could also display protective effects in other inflammatory disorders of the gastrointestinal tract, as mucositis in which dysbiosis have been implicated as key factor for inducing intestinal inflammation [41–45]. The medicament 5-FU commonly used in oncology generate as an adverse effect mucositis, causing weight loss to patients as around 80% of their epithelial cells population are destroyed by apoptosis thus leading to lower absorption of nutrients [46]. In our study, pretreatment with both *L. lactis* NZ9000 and *L. lactis*-PAP strains during 13 days had no influence on weight recovery of inflamed mice. Differently, Bowen and colleagues have shown that VSL#3 probiotic mixture could reduce weight loss in mice with mucositis after a long period of administration (28 days) [15]. Therefore, we believe that longer treatment duration is required to recover weight of mice injected with 5-FU.

A common feature of mucositis patients receiving 5-FU is the presence of inflammatory cells infiltrating into the *lamina propria* [2, 4]. Another characteristic of mucositis disorder is altered intestinal morphology, such as villus shortening and decreased villus/crypt ratio, with loss of the mucosal barrier integrity [7–11]. Thus, all this parameters were evaluated in this work in order to test the anti-inflammatory activity of both LL and LL-PAP strains. Histological analysis revealed that the administration of *L. lactis* NZ9000 in inflamed mice decreased the histopathological scores, with less polymorphonuclear infiltration and ulceration observed in samples. Surprisingly, PAP expression by *L. lactis* did not improve these parameters. Despite LL-PAP did not improve histopathological scores, morphometric analysis showed that it was able to better preserve intestinal villous architecture. Moreover, LL-PAP was able to preserve and improve Paneth cells activity, thus augmenting antimicrobial gastrointestinal function, as these cells are associated with the secretion of antimicrobial peptides. This was a very interesting result because PAP has also been reported to stimulate the proliferation of epithelial cells in the colon, including Paneth cells [47]. Mucositis is characterized by a very acute mucosal inflammation, in which the recruitment of neutrophils plays a major role on the pathophysiology process through the release of reactive oxygen species and inflammatory mediators [2]. Thus, we measured the activity of MPO enzyme, strictly produced by neutrophils, to estimate the cell influx into intestinal *lamina propria* from non-inflamed and inflamed mice receiving water, medium or LL and LL-PAP strains. Interestingly, we demonstrated that LL or LL-PAP treated mice reduced MPO activity after 5-FU injection. This result reveals the anti-inflammatory capacity of *L. lactis* NZ9000 strain to exempt neutrophil recruitment to the tissue. Moreover, recent work demonstrated that the increased influx of eosinophils is also an important event for the pathogenesis of mucositis [45]. Thus, we measured the activity of EPO enzyme, naturally produced by eosinophils, to estimate eosinophil influx into intestinal *lamina propria* across experimental groups. We showed that *L. lactis* NZ9000 strain expressing or not PAP was able to reduce eosinophil infiltration. A similar result was described by Holvoet colleagues [48] when another strain, *L. lactis* NCC2287, was used for the treatment of Eosinophilic esophagitis in mice. They showed that lactococci administration in mice significantly decreased esophageal eosinophilia, elicited by epicutaneous sensitization with *Aspergillus fumigatus* protein extract, reiterating the beneficial effects of *L. lactis* in another severe inflammatory disease [48].

Levels of secretory IgA were also determined as it is crucial to prevent pathogens to penetrate the epithelial barrier and, thus, to contain inflammatory processes [49]. We found that animals with mucositis that did not receive lactococci treatment had increased sIgA levels in the small bowel. This effect was expected, as the amount of this immunoglobulin increases during intestinal inflammatory process as a defense mechanism of the host [49, 50]. Interestingly, *L. lactis* NZ9000 administration decreased IgA secretion in mouse intestinal lumen. Again, no improvements were obtained with the use of *L. lactis* secreting PAP strain.

Altogether, our results have shown that *L. lactis* NZ9000 strain carrying pSEC vector without the cDNA of PAP was able to prevent 5-FU-induced intestinal inflammation in BALB/c mice. This was a very surprising and intriguing result because *L. lactis* NZ9000 strain is a derivative of *L. lactis* MG1363 used as starter cultures for cheddar cheese production [51, 52], and has not been reported as improving host health. Actually, few studies have reported beneficial effects of this species [48, 50, 53, 54]. Ballal and colleagues found that *L. lactis* I-1631 ameliorated colitis in T-bet−/− Rag2−/− mice and other two studies have demonstrated that either NCDO2118 sub. lactis or FC sub. cremoris presents anti-inflammatory properties in inflamed mice receiving chemical agent Dextran Sulphate Sodium (DSS) [48, 50, 53].

Furthermore, PAP expression by *L. lactis* subtly alleviate mucositis damage, as it did not show to decrease markers of inflammation such as, ulceration, pro-inflammatory cells infiltrate and IgA levels, but preserved architecture and increased secretory granules density inside Paneth cells in response to 5-FU inflammation.

## Conclusion

In conclusion, we have demonstrated that the allochthonous bacterium, *L. lactis* NZ9000, derived from dairy *L. lactis* MG1363 reveals to be a promising tool to prevent chemotherapy drug 5-Fluoracil-induced mucositis. Moreover, we opened the doors for future studies investigating possible factors involved in *L. lactis* NZ9000 anti-inflammatory effects. As beneficial effects has been demonstrated by the recombinant LL-PAP strain, further studies should be considered, such as biological confinement strategies preventing its dissemination into nature, in order to make it a safe approach to be tested in humans.

## Abbreviations

5-FU: 5-fluoracil
AMP: antimicrobial peptides
DNBS: dinitrobenzenosulfonic acid
EPO: eosinophil peroxidase
IBD: inflammatory bowel diseases
LAB: lactic acid bacteria
NICE: Nisin Control Expression System
PAP: pancreatitis-associated protein
sIgA: secretory immunoglobulin A
TFF-1: trefoil factor 1
